# Marine microalgae co-cultured with floc-forming bacterium: Insight into growth and lipid productivity

**DOI:** 10.7717/peerj.11217

**Published:** 2021-04-23

**Authors:** Chin Sze Yee, Victor Tosin Okomoda, Fakriah Hashim, Khor Waiho, Siti Rozaimah Sheikh Abdullah, Cosmas Alamanjo, Hassimi Abu Hasan, Emienour Muzalina Mustafa, Nor Azman Kasan

**Affiliations:** 1 Higher Institution Centre of Excellence (HICoE), Institute of Tropical Aquaculture and Fisheries, Universiti Malaysia Terengganu, Kuala Nerus, Terengganu, Malaysia; 2 Department of Fisheries and Aquaculture, Federal University of Agriculture Makurdi, Makurdi, Benue State, Nigeria; 3 Department of Chemical and Process Engineering, Faculty of Engineering and Built Environment, Universiti Kebangsaan Malaysia, Selangor Darul Ehsan, Malaysia; 4 Department of Agricultural Technology, Federal College of Forestry, Jos, Jos, Plateau, Nigeria; 5 Faculty of Food Science and Fisheries, Universiti Malaysia Terengganu, Kuala Nerus, Terengganu, Malaysia

**Keywords:** Pure strain, Microalgae-bacterium co-culture, Lipid content, Productivity

## Abstract

This study investigated the effect of co-culturing microalgae with a floc-forming bacterium. Of the six microalgae isolated from a biofloc sample, only *Thalassiosira weissflogii*, *Chlamydomonas* sp. and *Chlorella vulgaris* were propagated successfully in Conway medium. Hence, these species were selected for the experiment comparing microalgae axenic culture and co-culture with the floc-forming bacterium, *Bacillus infantis*. Results obtained showed that the co-culture had higher microalgae biomass compared to the axenic culture. A similar trend was also observed concerning the lipid content of the microalgae-bacterium co-cultures. The cell number of *B. infantis* co-cultured with *T. weissflogii* increased during the exponential stage until the sixth day, but the other microalgae species experienced a significant early reduction in cell density of the bacteria at the exponential stage. This study represents the first attempt at co-culturing microalgae with *B. infantis*, a floc-forming bacterium, and observed increased biomass growth and lipid accumulation compared to the axenic culture.

## Introduction

Microalgae are photosynthetic microorganisms with a biomass that is rich in lipids, proteins, and other compounds; it is also capable of surviving under harsh environmental conditions (Cuellar-Bermudez et al., 2015; Lee et al., 2017). Various microalgal species have attracted considerable attention in the past few decades because of their potential applications in biofertilizers, human and animal nutrition, pigment production (Milledge, 2011; Jiang et al., 2018; Koutra et al., 2018), biodiesel production (Zhu, Li & Hiltunen, 2016), and wastewater treatment (Christenson & Sims, 2011; Xia & Murphy, 2016; Quijano, Arcila & Buitrón, 2017). A microalgae-based approach to nutrition, energy production, and water treatment is not only considered to be economically sustainable but also an environmentally friendly alternative to conventional methods (Borowitzka, 2013; Gupta & Pawar, 2018). However, the low efficiency of conventional microalgae cultivation has restricted the development of the microalgae industry (Liu et al., 2019; Zhou et al., 2019).

In most algal monoculture systems, lipid accumulation occurs under nutrient-depleted conditions, but the stress conditions that induce increased storage of lipids also inhibit biomass productivity (Hannon et al., 2010; Contreras-Angulo et al., 2019; Mandal, Chanu & Chaurasia, 2020). Consequently, optimization of suitable conditions for producing both increased lipid and biomass may be challenging, as the culture conditions required for higher productivity of the two outcomes are contradictory (Hu et al., 2008; Markou & Nerantzis, 2013; Yang et al., 2018). Current efforts are therefore directed towards developing efficient production strategies that allow both rapid cell growth and lipid accumulation during the cultivation of microalgae (Chisti, 2013). One such production strategy is the co-culturing of microalgae with bacteria. Several studies have opined that microalgae-bacteria co-culture enhances both algal and bacterial culture (Thøgersen et al., 2018; Berthold et al., 2019; Zhou et al., 2020). This is because multiple relationships exist between the bacteria and microalgae which may be mutually beneficial, symbiotic, or parasitic (Ramanan et al., 2016; Cho et al., 2015). These varied biological interactions alter the metabolism of the microalgae-bacteria to meet one another’s needs in regulating nutrient mechanisms (Padmaperuma et al., 2018). For instance, the study by Han et al. (2016), reported that, in co-culture, the bacteria benefit from the oxygen and extracellular substances generated by microalgae while in return reimbursing them with carbon dioxide, vitamins, and other substances needed for optimum microalgal growth.

Microalgae are abundant in the natural environment and coexist with a wide range of microorganisms (Fuentes et al., 2016; Berthold et al., 2019). Symbiotic relationships between algae and microorganisms play important roles in natural ecosystems (Silva-Benavides & Torzillo, 2012), and have been well exploited in biofloc technology to improve the performance of aquaculture species (through reduction of the feed conversion ratio), wastewater treatment, and prevention of disease outbreaks (De Souza et al., 2014; Long et al., 2015; Ferreira et al., 2017; Wang et al., 2019; Nurarina et al., 2020). While studies have demonstrated the importance of different forms of bacterial/microalgae isolated from diverse environments (Gonzalez & Bashan, 2000; Mayali & Doucette, 2002; Kim et al., 2014; Fuentes et al., 2016; Toyama et al., 2018), only a few have reported the symbiotic effect of co-culturing floc-forming bacteria with microalgae dominant in biofloc (Wang et al., 2015).

We have earlier demonstrated that *Bacillus infantis* is more effective than *Nitratireductor aquimarinus* as a floc-forming bacterium under laboratory conditions (Harun et al., 2019). The reduction in flocculation time of biofloc with *B. infantis* has also been proposed as a “Rapid biofloc^®^ technology”, and a patent has been filed (Patent filed PI 2017703679). In this study, we isolated the microalgal species in biofloc samples collected from ponds used to raise Pacific Whiteleg shrimp, *Litopenaeus vannamei*. Thereafter, the symbiotic effect of co-culturing these microalgae with the floc-forming bacteria *B. infantis* was tested under laboratory conditions and compared with the axenic culture.

## Materials and Methods

### Isolation and identification of microalgae

Biofloc samples were collected from water in ponds used to culture Whiteleg shrimp, *L. vannamei*. Centrifugation and agar plate streaking techniques were used to obtain a pure culture of the microalgae species used in this study (Phang & Chu, 1999; Okomoda et al., 2021). In brief, the samples were washed four times by centrifugation at 9,000×*g* rpm for 15 min. After each wash, the supernatant was discarded and the residue (i.e. pellet) resuspended, filtered through CF/C filter paper, and resuspended using distilled water. This process was performed to separate the microalgal cells from undesirable particles and microorganisms (McCurdy & Hodgson, 1974; Badar, Yaakob & Timmiati, 2017; Berthold et al., 2019). Thereafter, Conway medium agar (Tompkins et al., 1995) was prepared and poured into sterilized Petri dishes (100 × 15 mm diameter). Using a sterilized pipette, the microalgae residue suspension was dropped into each Petri dish, then spread over the agar using a sterile platinum hook. The Petri dishes were placed in an upside-down position, sealed with Parafilm tape, and incubated for seven days under laboratory conditions. Samples of the colonies that formed were transferred to slides and observed under a compound microscope. Selection of the different colonies was according to their size and colour. Observed mixed cultures were sub-cultured (streaked) to obtain pure cultures of the different microalgal species.

Colonies were observed under a microscope to ensure there was no cross-contamination of the microalgal species. Isolates were identified following the method described by Hasle et al. (1997). The axenic microalgae colonies were then incubated in 20 mL of saltwater enriched with Conway medium; thereafter, they were sub-cultured in 50 and 100 mL medium. Incubation conditions were maintained at 24 °C, 30 ppt salinity, 12:12 light-dark photoperiod (1,200 Lux), and 24 h aeration. In addition, every two days the cultures were examined using a microscope to ensure there was no cross-contamination of microalgal species or bacteria contamination in the inoculum. When the exponential growth phase was attained, inoculation of the microalgae was performed for both the axenic and co-culture treatments.

### Experimental set-up and performance evaluation

Microalgae inoculum was produced for two treatments, namely; axenic single strain microalgae (i.e. control treatment) and axenic microalgae co-cultured with *B. infantis*. The *B. infantis* was obtained from the bioflocculation-bacteria collection at the Institute of Tropical Aquaculture and Fisheries (AKUATROP), University Malaysia Terengganu. To establish the axenic control treatment 50 mL of microalgae inoculum was mixed with 450 mL of sterilized saltwater in a 1 L Erlenmeyer flask. For the co-culture treatment, however, the inoculum ratio of microalgae to bacteria was determined at 9:1 (v/v); so the 1 L Erlenmeyer flask contained 450 mL of the sterilized saltwater, 45 mL of the microalgae inoculum, and five mL of the bacteria. The initial cell number of microalgae used ranged between 1.18 × 10^6^–2.0 × 10^6^ cell/mL. All the treatment groups were prepared in five replicates and incubated under the same environmental condition as described in “Isolation and Identification of Microalgae” (i.e. temperature = 24 °C; salinity = 30 ppt; photoperiod = 12:12 light-dark; photoperiod intensity = 1,200 Lux; and 24 h aeration).

To measure microalgae growth, the specific growth rate of microalgae was calculated based on concentration of chlorophyll a (Chl-a). A total of 10 mL samples were collected from the pure culture and co-culture experiment flasks and filtered through GF/C filter papers, followed by extraction in five mL acetone. Chl-a was thereafter quantified using a wavelength of 630, 645 and 665 nm in a UV-vis spectrophotometer (Strickland & Parsons, 1968). Biomass productivity, P (mg/L day), was calculated from the variation in Chl-a (mg/L) within the cultivation time. Specific growth rate (µ day^−1^) was calculated using the equation given by Latiffi et al. (2017), as shown below:

$${\rm Specific\; growth\; rate\; (\mu \; da}{{\rm y}^{ - 1}}{\rm )\; = \; }ln\; \left( {{X_1}/{X_0}} \right)/\left( {{t_1} - {t_0}} \right)$$

Where X_1_ and X_0_ represent the growth of algae (mg/L) at days t_1_ and t_0_ respectively.

To determine the biomass concentration in terms of dried weight, a 50 mL sample of microalgae in the growth medium from each replicate was washed twice with distilled water and filtered each time through CF/C filter paper (Allen, 1952; Droop, 1969; McCurdy & Hodgson, 1974). Thereafter it was dried in an oven at 105 °C for 24 h to constant weight This method has proven effective at separating microalgae cells from bacteria in co-culture experiments (Badar, Yaakob & Timmiati, 2017; Contreras-Angulo et al., 2019; Berthold et al., 2019; Zhou et al., 2020).

Lipid was extracted through a modification of the method of Bligh & Dyer (1959), according to the report of Phang et al. (2015). In brief, soon after determination of the biomass concentration (Badar, Yaakob & Timmiati, 2017), the sample was ground and homogenized in 2:1 methanol: chloroform solution. The mixture was then centrifuged, and the supernatant was collected. The process was repeated twice to extract any remaining fat from the mixture. Thereafter, the supernatant was mixed with two mL of chloroform and washed with two mL of distilled water by vortexing and centrifugation for 10 min. The lower layer of the mixture was collected and blow-dried under nitrogen gas. After drying, the sample was re-dissolved in one mL chloroform and blow-dried again. Blow-drying was repeated before the sample was cooled in a desiccator containing silica gel for 24 h. Lipid content (%) of each sample, based on dried cell weight, was estimated using an electronic balance. Microalgae lipid productivity (in mg/L day) was calculated only at the exponential stage of growth, by dividing the obtained lipid content by volume of culture and by the duration of cultivation. The choice of time for the evaluation of the lipid content was based on the results of an earlier optimization.

The density of *B. infantis* was also quantified during the experiment, following the method described by Han et al. (2016). In brief, one mL of the culture was collected from the experimental flask daily and diluted tenfold. One tenth (i.e. one mL) of the dilution was then mixed with 25 mL of TSA solid medium and cultured at 30 °C for 48 h. Plates with 30–300 colonies were then selected in order to calculate the bacterial density, based on colony-forming units (CFU).

### Statistical analysis

Descriptive statistics for Chl-a, specific growth rate, biomass concentration (DW), and lipid content were obtained using the software Minitab version 18.0. Statistical analysis of the parameters Chl-a, specific growth rate, and biomass concentration (DW) between treatments and days of cultivation were determined using two-way ANOVA and Tukey test at a significance level of 0.05 (Tybout et al., 2001). The mean differences of lipid content between the two treatments were analysed using the two sample t-test.

## Results

### Microalgae isolates used

**Figure 1:**
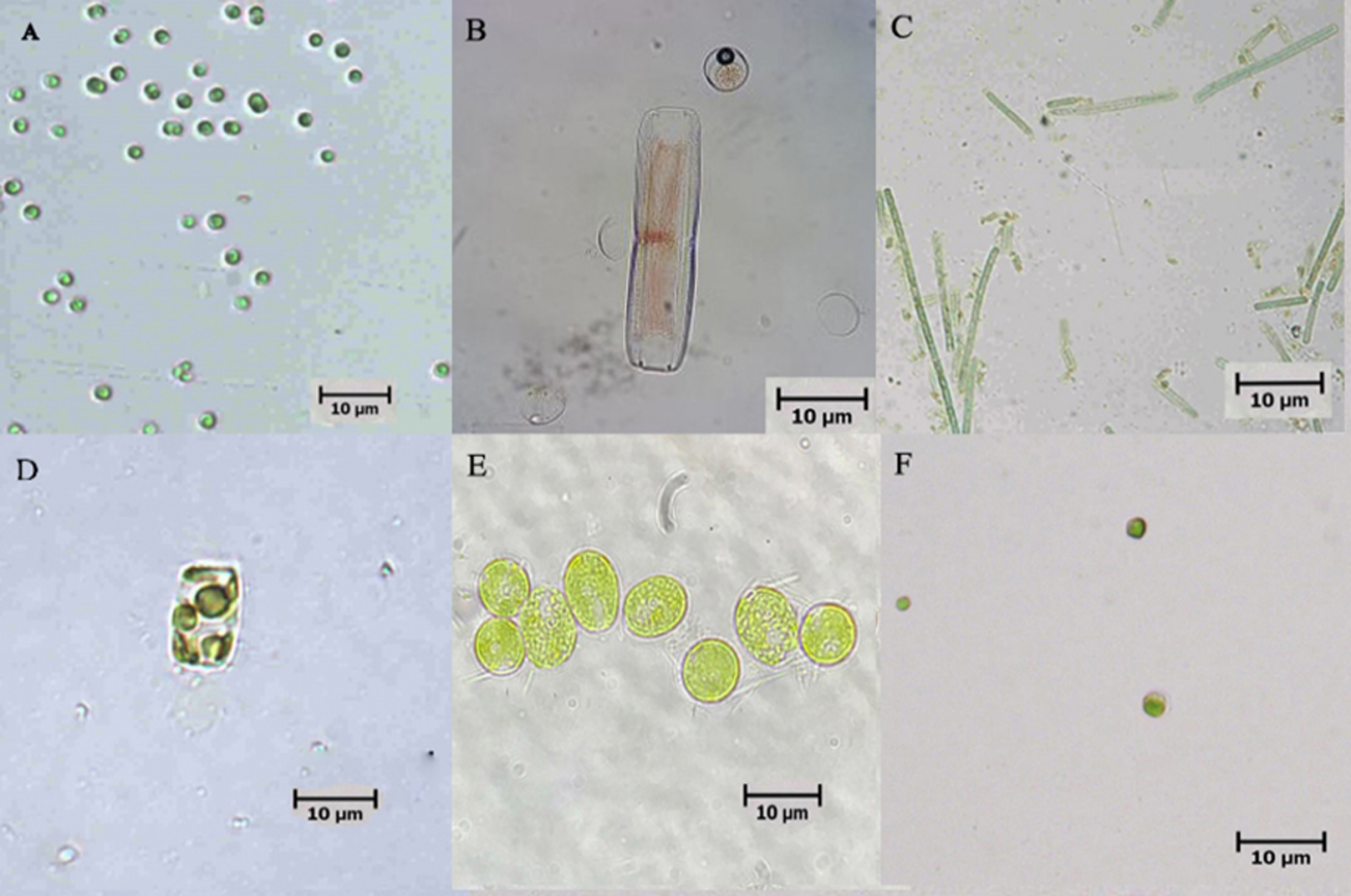
Microscopic pictures of microalgae isolated from biofloc-based system of Pacific whiteleg shrimp. (A) *Tetraselmis* sp.; (B) *Nitzschia* sp.; (C) *Oscillatoria* sp.; (D) *Thalassiosira weissflogii*; (E) *Chlamydomonas* sp.; (F) *Chlorella vulgaris*.

**Figure 2:**
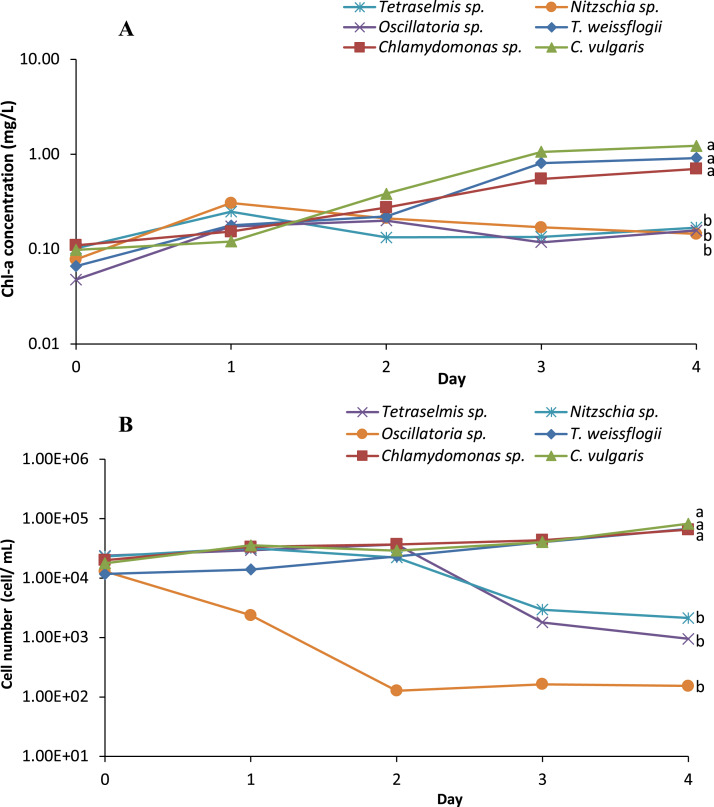
(A) Semi-logarithmic growth indicated by chorophyll a concentration and (B) cell number (cell/mL) of microalgae indicating cell density during inoculum producing process monitored in axenic condition.

Six microalgae strains were identified in the biofloc sample; they included *Tetraselmis* sp., *Nitzschia* sp., *Oscillatoria* sp., *Thalassiosira weissflogii*, *Chlamydomonas* sp. and *Chlorella vulgaris* (Fig. 1). As shown in Fig. 2, low Chl-a concentrations and a rapid decrease in cell density observed in *Tetraselmis* sp., *Nitzschia* sp. and *Oscillatoria* sp. resulted in decreased cell growth during the acclimatization phase. However, the growth performance of *T. weissflogii, Chlamydomonas* sp. and *C. vulgaris* was better in the medium supplemented with Conway enrichment, and optimum Chl-a concentration was maintained during the inoculum production phase. As a result, only these three microalgae were used for the axenic/co-culture experiment in this study.

### Growth of microalgae isolates in axenic condition and in co-culture

**Figure 3:**
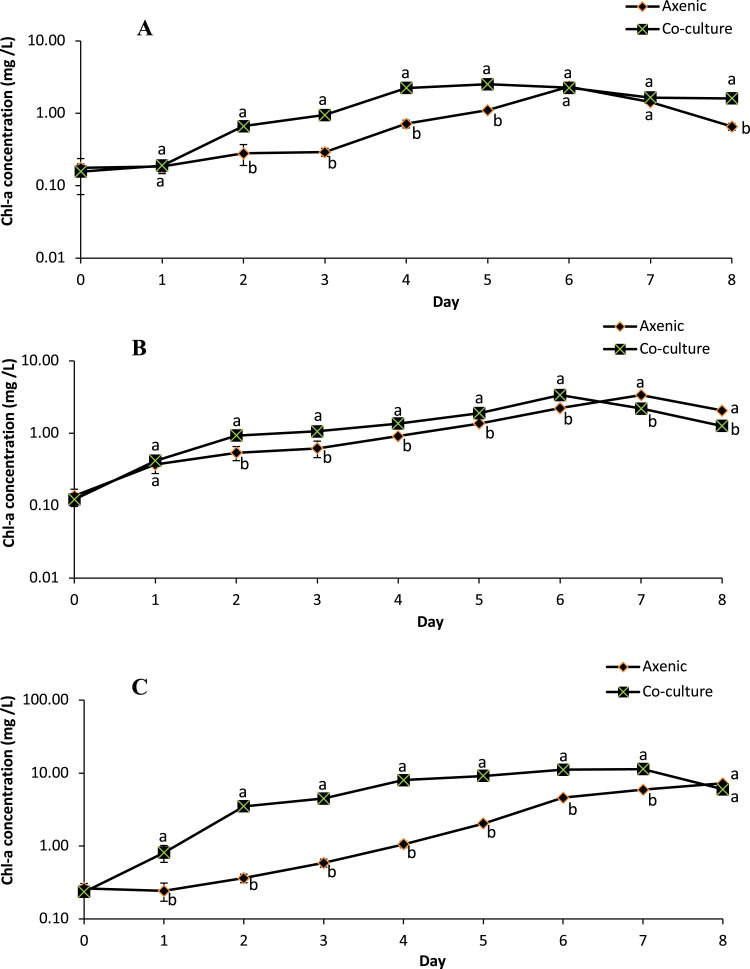
Semi-logarithmic growth curve of three different microalgae grouped cultivated in pure and co-culture treatments at 24 °C under 12:12 h light-dark cycle supplied with aeration until stationary stage (mean ± SD, *n* = 5). (A) *Thalassiosira weissflogii*; (B) *Chlamydomonas* sp.; (C) *Chlorellavulgaris*.

The growth profile of microalgae species in the axenic culture and in co-culture with *B. infantis* is presented in Fig. 3. Although the performance of *T. weissflogii* and *Chlamydomonas* sp. was similar in both treatments during the first two days, later a slight increase in Chl-a concentration was observed. For the *C. vulgaris* in the axenic condition, Chl-a concentration initially decreased (day 1), then recovered to a level above the initial concentration, and continued to increase until the sixth day. However, in co-culture conditions, Chl-a concentration of *C. vulgaris* was slightly reduced on day 3 and then increased afterward. In general, significantly higher growth was observed in the co-cultured microalgae species compared with those in axenic conditions (*p* ≤ 0.05).

The specific growth rate, as estimated between days 2 and 6 (for *T. weissflogii*) and between days 3 and 7 (for *Chlamydomonas* sp. and *C. vulgaris*), indicates the transition from an exponential growth stage to a stationary stage (Table 1). There were significant differences (*p* ≤ 0.05) in the specific growth rate of *T. weissflogii* between axenic and co-culture conditions; this indicates the occurrence of a rapid growth phase and achievement of peak (maximum) growth on days 4 and 2, respectively. *T. weissflogii* in axenic culture recorded a steady increase in biomass productivity up to day 6 (except day 5), however maximum biomass productivity of the co-culture treatment was achieved on day 4 (*p* ≤ 0.05). The specific growth rate of *Chlamydomonas* sp. in axenic and co-culture conditions achieved the maximal value on the sixth day. However, higher biomass productivity was observed in co-culture conditions compared to axenic conditions. *C. vulgaris* in co-culture conditions also attained maximum specific growth rate and maximum biomass productivity on the fourth day, before it started to decline.

**Table 1:** The specific growth rate (µ) and biomass productivity (P) of algae species.

Parameter	µ (day^–1^)		P (mg/L.day)	
	Axenic	Co-culture	Axenic	Co-culture
***T. weissflogii***				
2^nd^ day	0.13 ± 0.45*^d^	1.26 ± 0.15*^bc^	9.29 ± 4.37*^c^	31.73 ± 3.24*^c^
3^rd^ day	0.20 ± 0.09*^d^	0.36 ± 0.04*^b^	6.86 ± 1.59*^c^	19.10 ± 1.99*^d^
4^th^ day	0.90 ± 0.23*^a^	0.86 ± 0.06*^a^	28.52 ± 7.24*^b^	86.84 ± 4.67*^a^
5^th^ day	0.44 ± 0.15*^c^	0.12 ± 0.04*^c^	26.17 ± 7.84*^b^	69.05 ± 5.79*^b^
6^th^ day	0.76 ± 0.05*^b^	0.12 ± 0.05*^c^	82.96 ± 5.05*^a^	67.35 ± 6.65*^b^
***Chlamydomonas* sp.**				
3^rd^ day	0.34 ± 0.19*^c^	0.14 ± 0.06*^d^	12.89 ± 7.12*^d^	9.12 ± 4.42*^e^
4^th^ day	0.41 ± 0.27*^b^	0.25 ± 0.10*^c^	19.52 ± 9.95^d^	19.80 ± 7.88^d^
5^th^ day	0.40 ± 0.04*^b^	0.33 ± 0.03*^b^	30.36 ± 3.37^c^	36.21 ± 3.37^c^
6^th^ day	0.48 ± 0.07*^a^	0.57 ± 0.04*^a^	57.15 ± 7.73*^b^	98.68 ± 4.86*^a^
7^th^ day	0.42 ± 0.03*^b^	0.31 ± 0.01*^b^	77.48 ± 4.98^a^	79.08 ± 1.22^b^
***C. vulgaris***				
3^rd^ day	0.48 ± 0.23*^d^	0.25 ± 0.03*^b^	17.60 ± 4.51*^e^	65.85 ± 7.28*^c^
4^th^ day	0.59 ± 0.18^c^	0.58 ± 0.01^a^	31.36 ± 8.97*^d^	234.45 ± 5.30*^a^
5^th^ day	0.66 ± 0.11*^b^	0.13 ± 0.01*^c^	65.84 ± 8.67*^c^	76.54 ± 7.42*^c^
6^th^ day	0.81 ± 0.02*^a^	0.19 ± 0.01*^c^	170.93 ± 5.61*^a^	129.20 ± 6.70*^b^
7^th^ day	0.25 ± 0.01*^e^	0.02 ± 0.01*^d^	87.69 ± 5.82*^b^	76.44 ± 6.14*^c^

**Notes:**

* Value represents the mean of five replicate samples with a significant difference between treatments (*p* ≤ 0.05).

^a–e^Value represents the mean of five replicate samples with a significant difference of treatment against the day (*p* ≤ 0.05).

**Figure 4:**
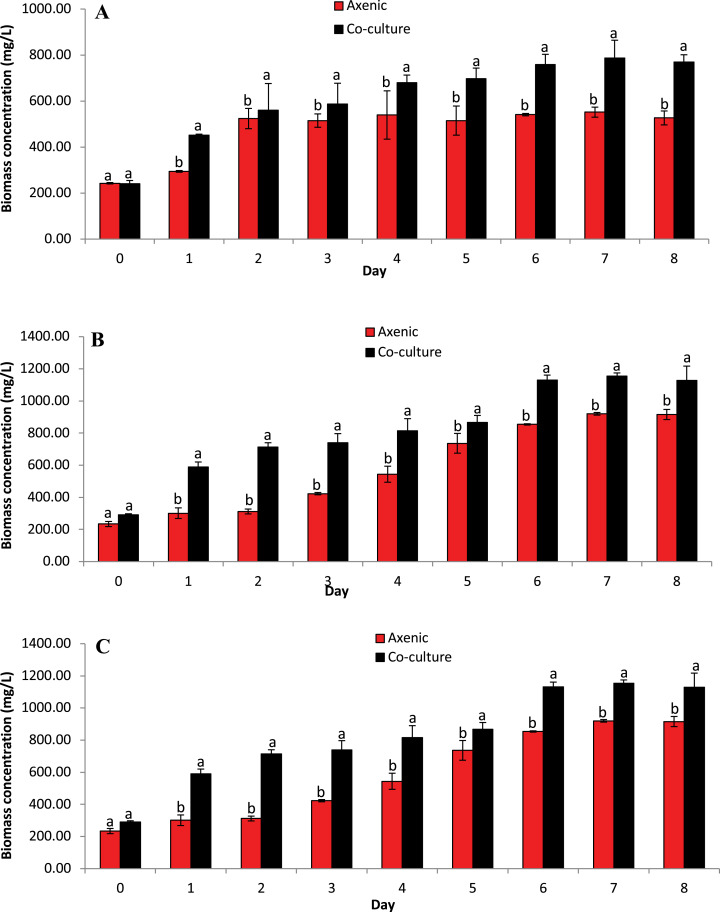
Biomass concentration based on dried microalgae cell weight. Comparison between microalgae in axenic condition and co-culture condition (mean ± SD, *n* = 5). (A) *Thalassiosira weissflogii*; (B) *Chlamydomonas* sp.; (C) *Chlorella vulgaris*.

The concentration of microalgae biomass is given by dry weight in Fig. 4. The biomass concentrations of microalgae obtained in the co-culture treatment was 20.32% higher than the biomass concentration obtained in axenic microalgae (*p* ≤ 0.05). These data indicate that the selected microalgae grew better in co-culture with *B. infantis* than in axenic conditions. Furthermore, *T. weissflogii* grown in co-culture showed a noticeable increase from day 4 to day 8 (*p* ≤ 0.05), compared to the axenic condition (Fig. 4A). Also, the maximum biomass concentration of *Chlamydomonas* sp. was observed on the fifth and seventh day for the axenic condition and co-culture condition, respectively (Fig. 4B). The biomass concentration of *C. vulgaris*, in contrast, increased steadily until the eighth day in both the co-culture and axenic treatments (Fig. 4C).

### Lipid content of microalgae isolates in axenic condition and in co-culture

Microalgae lipid content was evaluated when the microalgae experienced the highest specific growth rate; hence, the lipid content of the microalgae at growth climax was evaluated as well as that after the climax point, as presented in Table 2. A decreasing trend of lipid content, post-growth climax, was observed in axenic conditions, while the converse was observed for the co-culture treatment (except for *C. vulgaris*). A similar trend was observed in the lipid productivity of the microalgae in this study. However, the biomass productivity of *Chlamydomonas* sp. was not significantly different among treatment and cultivation days.

**Table 2:** Lipid content based on dry cell weight and lipid productivity of algae species.

Treatment	Day of cultivation	Lipid content (%)	Lipid productivity (mg/L.day)
***T. weissflogii***			
Axenic	4^th^	4.49 ± 0.01*	1.69 ± 0.32*
	5^th^	3.59 ± 0.003*	0.72 ± 0.06*
Co-culture	4^th^	4.11 ± 0.006*	1.00 ± 0.09*
	5^th^	5.98 ± 0.01*	1.13 ± 0.03*
***Chlamydomonas* sp.**			
Axenic	6^th^	5.74 ± 0.01*	1.36 ± 0.11
	7^th^	4.20 ± 0.005*	0.84 ± 0.09
Co-culture	6^th^	3.17 ± 0.009*	0.79 ± 0.22*
	7^th^	5.94 ± 0.02*	1.34 ± 0.06*
***C. vulgaris***			
Axenic	6^th^	5.00 ± 0.02	1.51 ± 0.09
	7^th^	3.50 ± 0.035	0.70 ± 0.19
Co-culture	4^th^	4.66 ± 0.047	1.17 ± 0.21
	5^th^	3.67 ± 0.002	0.56 ± 0.08

**Note:**

* Value represents mean of five replicate samples with significant difference (*p* ≤ 0.05).

### Bacterial density in co-cultured microalgae species

**Figure 5:**
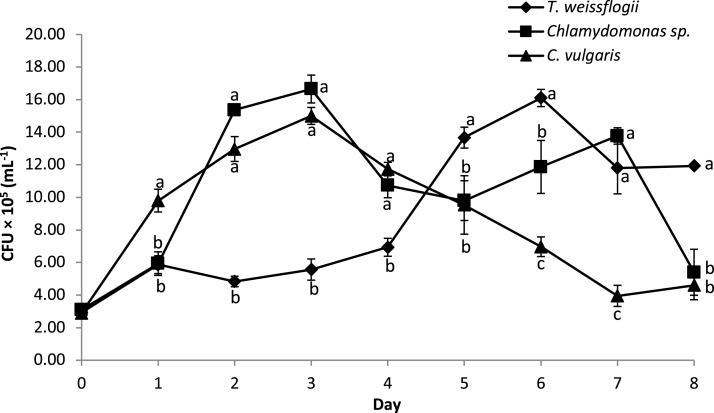
Growth performance of *B. infantis* co-cultured with different microalgae species (mean ± SD, *n* = 5).

The bacterial density of *B. infantis* in the co-cultured treatment is given in Fig. 5. The result showed significant variation in the cell density over time across the different microalgae tested. Peak CFU for *B. infantis* was observed in the co-cultured treatment with *Chlamydomonas* sp. and *C. vulgaris* on the third day of culture. Co-cultured *T. weissflogii*, on the other hand, consistently had a low number of CFU up until the fifth day when it started recording the highest values of all the treatments, until the eighth day (except for day 7, where performance was at the median level).

## Discussion

Berthold et al. (2019) stressed the importance of considering indigenous algal species when choosing microalgae species for culture, as they are already adapted to local climatic and aquatic conditions. This is necessary to avoid any concerns regarding the introduction of invasive species to the environment, as is common with the introduction of non-native species of microalgae. In this study native algae were isolated from mature biofloc water and used for the axenic and co-culture experiment. The six strains were isolated morphologically and ranged from unicellular organisms to filamentous forms that correspond to the classes of Chlorophyceae, Bacillariophyceae, and Cynaophyceae, in line with the observations of Manan, Moh & Kasan (2017). Of the microalgae that were isolated and supplemented with Conway enrichment medium, only three (namely *T. weissflogii*, *Chlamydomonas* sp. and *C. vulgaris*) did not experience any inhibitory effect on growth during the inoculum and production process. Although the reason for this is not well understood, it is hypothesized that this may be linked to different levels of auxin synthesis by the different microalgae, which would influence cell division and the metabolism of the microalgal species (Piotrowska-Niczyporuk & Bajguz, 2014; Stirk et al., 2013).

Nitrogen is an essential component of Chl-a and Chl-b in marine phytoplankton, as it has a significant influence on photosynthesis (Ördög et al., 2012). From the optimum Chl-a content and biomass observed in this study, it can be concluded that the organic compounds in the media were sufficient to support the cell growth of the microalgae. This study observed higher levels of growth in the co-cultured microalgae than in the axenic treatment. This is similar to the results reported in a study by Toyama et al. (2018) on *Chlamydomonas reinhardtii*, *C. vulgaris* and *Euglena gracilis* co-cultured with indigenous bacteria in wastewater effluent. Earlier studies by Kim et al. (2014) also found that plant phycosphere symbionts, such as Rhizobium, have growth-promoting effects on algae. While a symbiotic system of *Azospirillum* sp. and microalgae, *Chlorella* sp., in an artificial medium was reported to effectively increase the productivity of the microalgae cells (Gonzalez & Bashan, 2000). Fuentes et al. (2016) had earlier hypothesized that symbiotic associations in the bacteria-microalgae community may influence the growth of microalgae with effects ranging from promoting to slowing down growth. This was demonstrated in the study by Mayali & Doucette (2002), who reported that certain kinds of bacteria in the commensal bacterial community of the dinoflagellate *Karenia brevis*, successfully competed against algae, killing bacteria 41-DBG2 for nutrients, hence, protecting the growth of the microalgae by its algicidal bacteria attack.

This study has shown that the floc-forming bacteria *B. infantis* is likely part of the community of microalgae growth-promoting bacteria (MGPB), based on the observed increase in Chl-a concentration and biomass productivity, as well as specific growth rate. This is similar to the opinion of Wang et al. (2015) on the optimized performance of *C*. *vulgaris* co-cultured with the bioflocculant-producing bacteria F2, *Rhizobium radiobacter* in synthetic wastewater. Although the mechanism underlying the improved performance in the current study is not yet understood, *Bacillus* are known to be aerobic bacteria that are involved in nutrient exchange in the aquatic environment (Su et al., 2011). Bacteria remineralization has been reported to account for a large portion of the assimilated organic compounds in phytoplankton (Tai et al., 2009). In the study by Amavizca et al. (2017), it was observed that plant growth-promoting bacteria affect microalgae biomass concentration and lipid content remotely through gas and volatile organic compounds exchange. Therefore, since a positive correlation exists between photosynthesis and microbial activity, the co-culture system in this study must have supported microalgae photosynthesis and biomass accumulation better than that observed in the axenic culture system. It is also important to state that the maximum growth of microalgae was achieved earlier in the co-culture treatment, thereby shortening the production period of the microalgae. In addition to the advantage of reducing the risk of culture contamination, Padmaperuma et al. (2018) also discussed the possibility of reduced operational time and cost with microalgae-bacteria co-culture.

The lipid content of axenic microalgae in this study was observed to be higher at the peak of cell growth compared with the bacteria co-culture treatment. However, these values were significantly higher post the peak of growth for the co-culture treatment of *T. weissflogii* and *Chlamydomonas* sp. and significantly reduced in the axenic treatments. Contrary to the finding for *C. vulgaris* in this study, Rajapitamahuni et al. (2019) reported higher lipid productivity from co-cultivation of *Chlorella variabilis* with bacteria *Idiomarina loihiensis*. An earlier study had demonstrated a higher accumulation of lipids, protein, and fatty acid in microalgae grown under nutrient stress conditions (Roleda et al., 2013; Rajapitamahuni et al., 2019). The low lipid yield observed during the growth phase in the current study for co-culture microalgae therefore may be an indication that they were not nutrient deficient (Lee et al., 2014; Griffiths & Harrison, 2009). Nevertheless, the subsequent increase after the delay in lipid accumulation may suggest that *T. weissflogii* and *Chlamydomonas* sp. channelled most of their energy into photosynthetic activity before the cells shifted their biosynthetic pathway into lipid accumulation as energy storage reserves.

Besides the better performance of microalgae, this study also observed that bacteria concentration was affected in the microalgae-bacteria co-culture. The performance trend suggests that the density of *B. infantis* increased as the growth rate of the microalgae decreased. This was most likely an effect of the excretion of extracellular substances by the different microalgae, which play an important role in the increase or decline in bacteria density (Han et al., 2016; Guo & Tong, 2014). In summary, the current study suggests that the three microalgae strains supplied the organic carbon needed to effectively support bacterial growth. This is in line with the finding of Zhou et al. (2020) on the growth and density of algal and bacterial co-cultures.

## Conclusion

Production strategies that facilitate both increased biomass and higher lipid accumulation are key to achieving the economic viability of algae production. This study has demonstrated that co-culturing floc-forming bacterial *B. infantis* with *T. weissflogii* and *Chlamydomonas sp*. increased biomass and lipid content, while only affecting biomass productivity in *C. vulgaris*. This observation indicates that different species of microalgae may respond differently to co-culturing with bacteria. Hence, there is a need to screen and experimentally test more microalgae in different co-culture systems with other floc-forming bacteria. The higher accumulation of biomass and lipid concentration, in a relatively shorter time, may partly explain the early flocculation and better performance observed with ponds bio-augmented with *B. infantis* in our earlier reported studies on the Rapid biofloc^®^ technology.

## Supplemental Information

10.7717/peerj.11217/supp-1 Supplemental Information 1 Data set 1.

10.7717/peerj.11217/supp-2 Supplemental Information 2 Data set 2.

10.7717/peerj.11217/supp-3 Supplemental Information 3 Data set 3.

10.7717/peerj.11217/supp-4 Supplemental Information 4 Data set 4.

10.7717/peerj.11217/supp-5 Supplemental Information 5 Data set 5.

10.7717/peerj.11217/supp-6 Supplemental Information 6 Data set 6.

10.7717/peerj.11217/supp-7 Supplemental Information 7 Data set 7.

## Additional Information and Declarations

### Data Availability

The following information was supplied regarding data availability: The raw data for the article are in the Supplementary Files. These data describe the collection for axenic and co-culture treatments.
